# Successful biliary biopsy in a patient with surgically altered anatomy using a slim peroral cholangioscope via an endoscopic ultrasound-guided biliary drainage fistula

**DOI:** 10.1055/a-2462-1757

**Published:** 2024-11-22

**Authors:** Noriyuki Hirakawa, Takayoshi Tsuchiya, Ryosuke Tonozuka, Shuntaro Mukai, Kenjiro Yamamoto, Takao Itoi

**Affiliations:** 1 13112 Gastroenterology and Hepatology, Tokyo Medical University, Shinjuku-ku, Japan

Endoscopic retrograde cholangiopancreatography (ERCP) can be performed in patients with surgically altered anatomy using a balloon-assisted enteroscope. However, postoperative adhesions and unique anatomical characteristics result in lower technical success rates, ranging from 75.8% to 94% 1 2 3 . Recently, endoscopic ultrasound-guided biliary drainage (EUS-BD) has been used after unsuccessful transpapillary biliary drainage attempts 4 5 . Nevertheless, obtaining a biopsy through an EUS-BD fistula remains technically challenging. This report presents a case where distal cholangiocarcinoma was diagnosed macroscopically and pathologically using a slim peroral cholangioscope (eyeMAX; Micro-Tech Co., Ltd., Tokyo, Japan) via an EUS-BD fistula.

A 66-year-old man with a history of Roux-en-Y reconstruction following gastric cancer resection presented with obstructive jaundice. Abdominal contrast-enhanced computed tomography revealed a stricture with circumferential wall enhancement in the distal bile duct ( Fig. 1 **a** ). Balloon endoscopy-assisted ERCP was attempted; however, adhesions prevented enteroscope insertion into the major papilla ( Fig. 1 **b** ). Consequently, EUS-BD was attempted. The bile duct was punctured with a 22-gauge needle. Cholangiography confirmed a distal bile duct stricture. A 7-Fr dedicated plastic stent was inserted through the fistula ( Fig. 1 **c, d** ). Considering the anticipated difficulty of the EUS-guided rendezvous technique due to adhesions, we attempted a biopsy via the EUS-BD fistula.

**Fig. 1:**
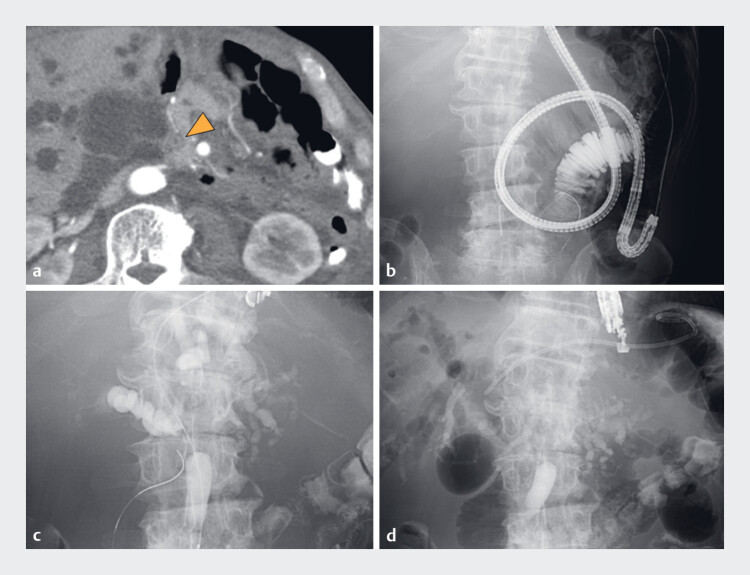
**a** Abdominal contrast-enhanced computed tomography showing a stricture (arrowhead) in the distal bile duct and a small, high-attenuation mass encircling the duct. **b** Balloon-assisted enteroscope failed to reach the papilla. **c** Fluoroscopic image showing a stricture in the distal bile duct. **d** A 15-cm 7-Fr dedicated plastic stent was inserted through the fistula.

One month later, we dilated the fistula using an ERCP catheter passed over the 7-Fr stent, allowing easy insertion of a 3.2-mm cholangioscope without additional balloon catheter dilation ( Video 1 ). The cholangioscope revealed a pinhole stricture with abnormal vascular proliferation in the distal bile duct ( Fig. 2 **a** ). Micro biopsy forceps were used to obtain specimens from the stricture ( Fig. 2 **b, c** ). The position of the stricture was confirmed fluoroscopically. Following cholangioscope withdrawal, additional biopsy specimens were acquired using an ERCP guide sheath (Olympus Medical, Tokyo, Japan) ( Fig. 2 **d, e** ). No procedure-related adverse events occurred. Both biopsy specimens indicated adenocarcinoma, and surgical intervention was scheduled ( Fig. 2 **f** ).

Biliary biopsy was successfully performed in a patient with surgically altered anatomy using a slim peroral cholangioscope through an endoscopic ultrasound-guided biliary drainage fistula.Video 1

**Fig. 2:**
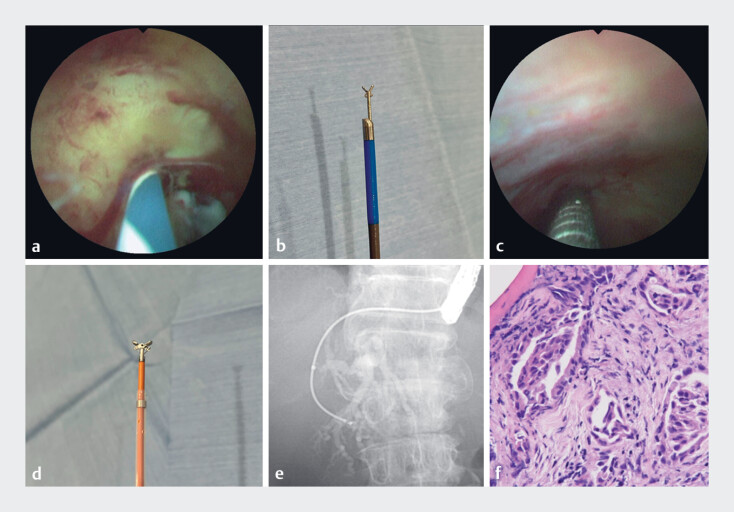
**a** The distal bile duct was pinhole-shaped and had abnormal vascular proliferation. **b** Biopsy forceps used with the slim peroral direct digital cholangioscope (eyeMAX; Micro-Tech Co., Ltd., Tokyo, Japan). **c** The stricture site was identified endoscopically, and a biopsy was performed. **d** The biopsy forceps were deployed through an endoscopic retrograde cholangiopancreatography guide sheath (Olympus Medical, Tokyo, Japan). **e** Biopsy specimens were obtained from the stricture site under fluoroscopic guidance. **f** Biopsy specimens showed adenocarcinoma.

Endoscopy_UCTN_Code_TTT_1AS_2AH

## Notes

Endoscopy E-Videos https://eref.thieme.de/e-videos
*E-Videos* is an open access online section of the journal *Endoscopy* , reporting on interesting cases and new techniques in gastroenterological endoscopy. All papers include a high-quality video and are published with a Creative Commons CC-BY license. Endoscopy E-Videos qualify for HINARI discounts and waivers and eligibility is automatically checked during the submission process. We grant 100% waivers to articles whose corresponding authors are based in Group A countries and 50% waivers to those who are based in Group B countries as classified by Research4Life (see: https://www.research4life.org/access/eligibility/ ). This section has its own submission website at https://mc.manuscriptcentral.com/e-videos .

## References

[1] Yamauchi H Kida M Okuwaki K Short-type single balloon enteroscope for endoscopic retrograde cholangiopancreatography with altered gastrointestinal anatomy World J Gastroenterol 2013 19 1728 1735 23555161 10.3748/wjg.v19.i11.1728 PMC3607749

[2] Anvari S Lee Y Patro N Double-balloon enteroscopy for diagnostic and therapeutic ERCP in patients with surgically altered gastrointestinal anatomy: a systematic review and meta-analysis Surg Endosc 2021 35 18 36 32789590 10.1007/s00464-020-07893-x

[3] Tanisaka Y Ryozawa S Mizuide M Status of single-balloon enteroscopy-assisted endoscopic retrograde cholangiopancreatography in patients with surgically altered anatomy: systematic review and meta-analysis on biliary interventions Dig Endosc 2021 33 1034 1044 33073407 10.1111/den.13878

[4] Itoi T Sofuni A Itokawa F Endoscopic ultrasonography-guided biliary drainage J Hepatobiliary Pancreat Sci 2010 17 611 616 10.1007/s00534-009-0196-1 19806298

[5] Mukai S Itoi T Sofuni A EUS-guided antegrade intervention for benign biliary diseases in patients with surgically altered anatomy (with videos) Gastrointest Endosc 2019 89 399 407 30076841 10.1016/j.gie.2018.07.030

